# Supplementary figures and images for: Metabolomics of developmental changes in Triatoma sanguisuga gut microbiota
Source: PLoS One. 2023 Feb 24;18(2):e0280868. doi: 10.1371/journal.pone.0280868 (PMC9955940; doi:10.1371/journal.pone.0280868)

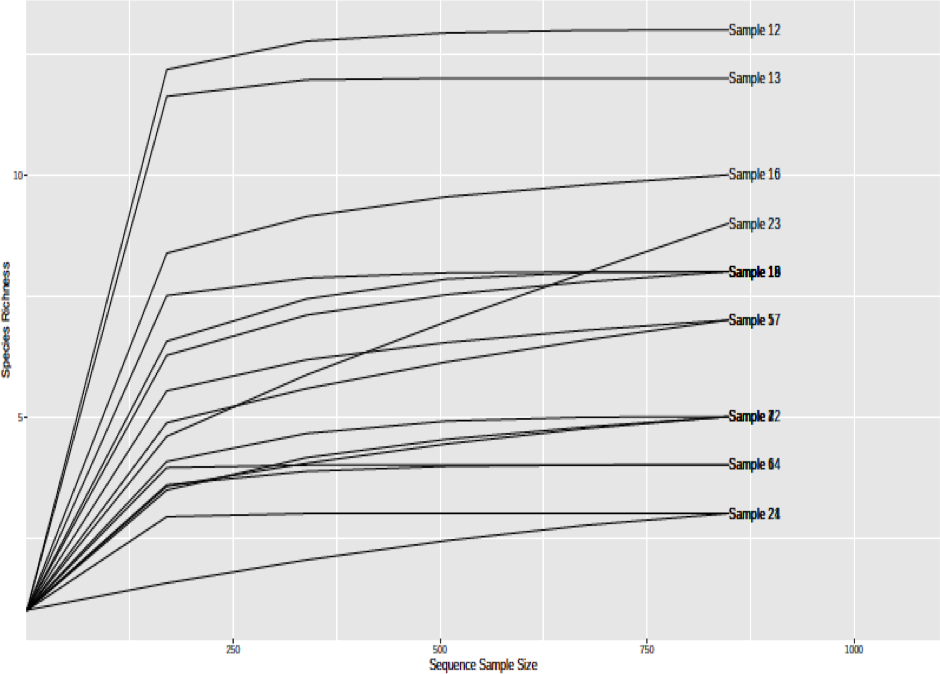

Supplement: S1 Fig — Curves indicate adequate sequencing depth for samples included in the analysis. (TIF) [file pone.0280868.s001.tif]

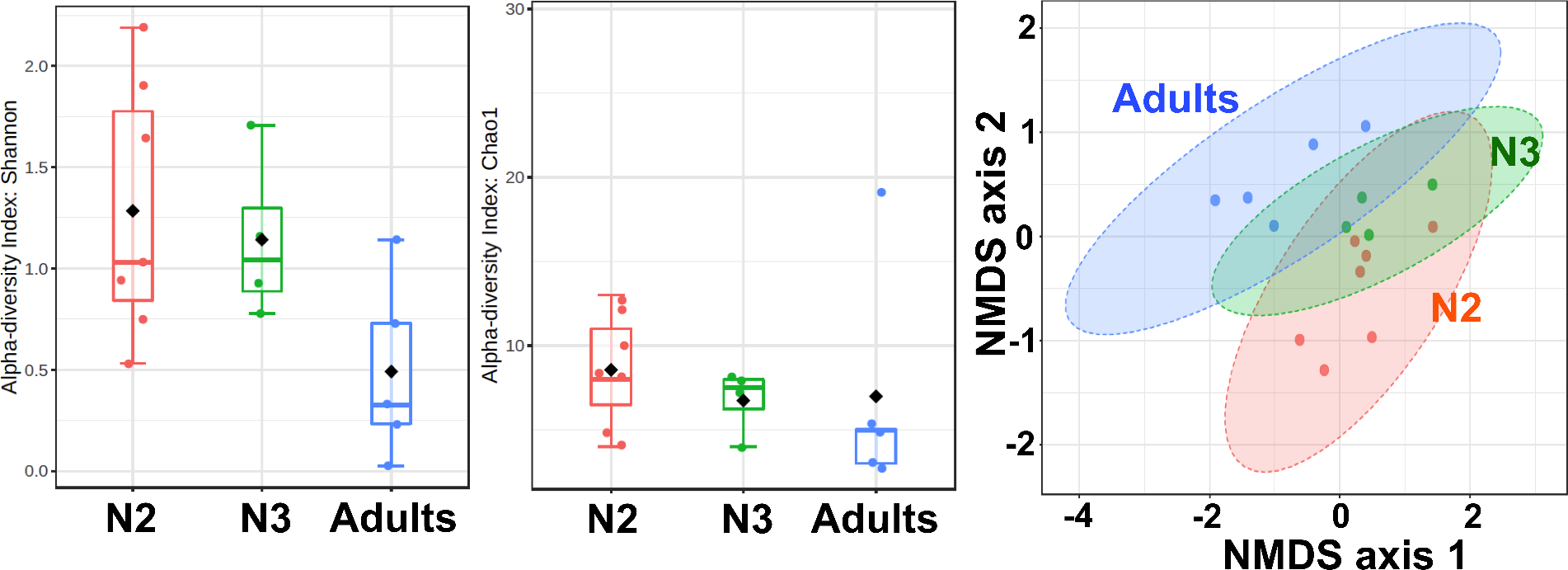

Supplement: S2 Fig — For alpha diversity, there was no significant difference in Shannon (t = 0.45, P = 0.65) and Chao1 indices (t = 1.34, P = 0.21) between second (N2) and third stage nymphs (N3). Beta diversity was also not different between N2 and N3 (PERMANOVA, F = 1.9; P = 0.09). (TIF) [file pone.0280868.s002.tif]
